# Potential Biomarkers of Dysmenorrhea Relief: A MEG Study of Hinoki Aromatherapy and Working Memory

**DOI:** 10.3390/biomedicines12102189

**Published:** 2024-09-26

**Authors:** Pei-Li Chou, Han-Sheng Huang, Chien-An Yao, Li-Min Wang, Jen-Jie Chieh, Bai-Chuang Shyu, Shu-Hsien Liao, Chiang-Ting Chien

**Affiliations:** 1 Department of Family Medicine, National Taiwan University Hospital, Taipei 100, Taiwanyao6638@gmail.com (C.-A.Y.); 2 Department of Life Science, National Taiwan Normal University, Taipei 11677, Taiwan; 3 Institute of Electro-Optical Engineering, National Taiwan Normal University, Taipei 11677, Taiwan; hansheng9527@gmail.com (H.-S.H.); jjchieh@ntnu.edu.tw (J.-J.C.); 4 Department of Physics, National Taiwan University, Taipei 106, Taiwan; liminwang@ntu.edu.tw; 5 Institute of Biomedical Sciences, Academia Sinica, Taipei 11529, Taiwan; bmbai@gate.sinica.edu.tw

**Keywords:** MEG, aromatherapy, dysmenorrhea, working memory

## Abstract

**Background/Objectives**: This study explored the potential of Hinoki aromatherapy to induce biomarkers of dysmenorrhea relief through working memory. Structural magnetic resonance imaging and magnetoencephalography (MEG) were used to examine their effects on neurophysiological responses to a visual working memory (VWM) test. Behavioral performance was measured to understand its effects on the overall working memory. **Methods**: Twenty-four healthy participants completed the VWM task during nonmenstruation and menstruation. Behavioral (accuracy and reaction time) and neurophysiological (event-related fields, source estimation, and permutation *t*-test on source data) measures were assessed without and with Hinoki aromatherapy. **Results**: A significant difference in the ratio of accuracy to reaction time was found without and with aromatherapy in participants with dysmenorrhea, suggesting that aromatherapy may improve working memory performance in this population. MEG analysis revealed high temporal resolution of evoked latency and intensity during the VWM task. Source localization of the activation aimed to identify brain areas involved in dysmenorrhea. Aromatherapy reduced signals in these areas, which may also contribute to reducing dysmenorrhea-related visual signals. **Conclusions**: Based on these findings, Hinoki aromatherapy may be a promising treatment option for alleviating dysmenorrhea and improving related symptoms by reducing activity in brain pain processing regions. These regions include the left entorhinal cortex, inferior temporal gyrus, primary visual cortex, retrosplenial cortex, and presubiculum. Furthermore, decreased activity in these areas with aromatherapy suggests that they could be used as biomarkers of dysmenorrhea relief.

## 1. Introduction

Dysmenorrhea, characterized by lower-abdominal pain during menstruation (MC), affects an unknown but significant number of women. Systematic reviews estimate its prevalence from 16% to 95% [1]. A study using high-resolution T1-weighted anatomical brain magnetic resonance imaging (MRI) found that primary dysmenorrhea (PDM) is associated with a high prevalence of normal variants but no brain abnormalities [2]. Menstrual pain, characterized by short-lasting cyclic pain, is associated with structural changes in the brain that are trait-related and rapid-state-related [3,4]. In women with PDM, a brain region called the periaqueductal gray matter (PAG) shows increased communication (hyperconnectivity) with sensory and motor cortices during painful periods. However, this same region displays decreased communication (hypoconnectivity) with the dorsolateral prefrontal cortex (PFC) and the default mode network (including the ventromedial PFC, posterior cingulate cortex, or posterior parietal cortex) throughout their menstrual cycle, including MC and ovulation [5]. Brain-derived neurotrophic factor (BDNF) plays a role in pain modulation within the PAG, and the BDNF Val66Met polymorphism contributes toward susceptibility to PDM with diverse functional expressions of the descending pain modulatory systems (DPMS) [6].

Working memory, which refers to the cognitive system that allows individuals to temporarily store and process information, is fundamental to various cognitive functions, including language comprehension, learning, and overall intelligence [7]. In the late follicular phase, increased brain activity in the left frontal circuitry is associated with higher estradiol levels and decreased working memory performance. Conversely, increased activation in right hemisphere regions is associated with improved task performance [8]. Scientists traditionally use long-lasting brain signals, called event-related potentials (ERPs) in electroencephalography (EEG) recordings and event-related fields (ERFs) in magnetoencephalography (MEG) recordings, to investigate cognitive processes. These signals are obtained by averaging the ongoing brain activity (EEG or MEG) in response to specific events. ERPs and ERFs, which are linked to mental activities, typically peak a few hundred milliseconds after the event occurs and arise from areas of the brain cortex involved in integrating information [9,10]. MEG is a noninvasive brain imaging technique that offers good spatial resolution and excellent temporal resolution, making it well suited for studying the brain activity of neurologically normal participants. The latency and dipole intensity and localization of M170, an important neural component and signal associated with cognitive functions such as working memory, is a stable parameter [11]. α-wave (8–12 Hz, low amplitude), which occurs with an awake but relaxed condition, has also been recorded in dysmenorrhea participants by MEG.

Aromatherapy is an alternative method to relieve the discomfort during the MC period [12,13]. For example, lavender has antispasm, sedation, and relaxing effects, and chamomile has anti-inflammation and antidepression effects. Clary sage and rose are also beneficial for dysmenorrhea [14]. Aromatherapy has been used traditionally as a nonpharmacological method to reduce the symptoms of dysmenorrhea, relieve uterine cramps, and decrease pain and anxiety [13,15]. Olfactory inhalation of Hinoki oil decreased systolic pressure and heart rate, and participants had pleasant feelings and felt physiologically relaxed [16,17]. In a study of 13 female university students (mean age, 21.5 ± 1.0 years), olfactory stimulation with Hinoki cypress leaf oil significantly reduced oxyhemoglobin concentration in the right PFC and increased parasympathetic nervous activity [17].

Emerging research suggests that specific brain regions are promising biomarkers of dysmenorrhea relief. Several studies have highlighted the involvement of different areas in pain processing and its emotional aspects. The left entorhinal cortex (ERC) and inferior temporal gyrus (ITG) may be implicated in physiological pain processing, with their activity positively correlated with pain intensity in women with dysmenorrhea [18]. The activity of these brain regions, specifically the ERC, may also be influenced by central sensitization factors that can amplify pain perception. Evidence suggests that in women with endometriosis, a condition often co-occurring with chronic pelvic pain, central sensitization is mediated by the upregulation of PAG and increased ERC activity. Furthermore, anxiety amplifies pain through increased ERC activity, and depression enhances the emotional component of pain [18]. The primary visual cortex (PVC) is a potential candidate due to its established role in DPMS in migraine [19]. This system is also linked to PDM, highlighting a possible connection between dysmenorrhea and PVC [5]. The shared pathophysiology of menstrual migraine and dysmenorrhea, involving prostaglandin overproduction, further strengthens this connection, considering the high co-occurrence of these conditions [20]. Beyond pain processing, the retrosplenial cortex (RSC) and presubiculum (PreS) also hold promise. RSC shows a positive correlation with pain intensity, whereas PreS acts as a bridge between the hippocampus and amygdala, relaying processed information through ERC and suggesting its role in processing emotional memories associated with PDM [5,21].

This study aimed to explore the potential of five brain regions—left ERC, ITG, PVC, RSC, and PreS—as biomarkers of dysmenorrhea relief. By employing structural MRI (sMRI) and MEG alongside a visual working memory (VWM) task, this study investigated the effects of Hinoki aromatherapy on behavioral performance (accuracy and reaction time) and neurophysiological responses (ERF of working memory). To analyze the changes in brain activity induced by Hinoki aromatherapy during the VWM task, this study utilized dynamic statistical parametric mapping (dSPM), implemented using the MNE software (v1.2.3) [22], to estimate the spatial and temporal distribution of the effects of aromatherapy on brain activity. Specifically, this study used permutation *t*-tests on source data with spatiotemporal clustering [23], also available in MNE, to identify the specific brain regions and time points most affected by aromatherapy and further explore the underlying mechanisms of action.

## 2. Materials and Methods

### 2.1. Protocol and Participants for Working Memory

**Figure 1:**
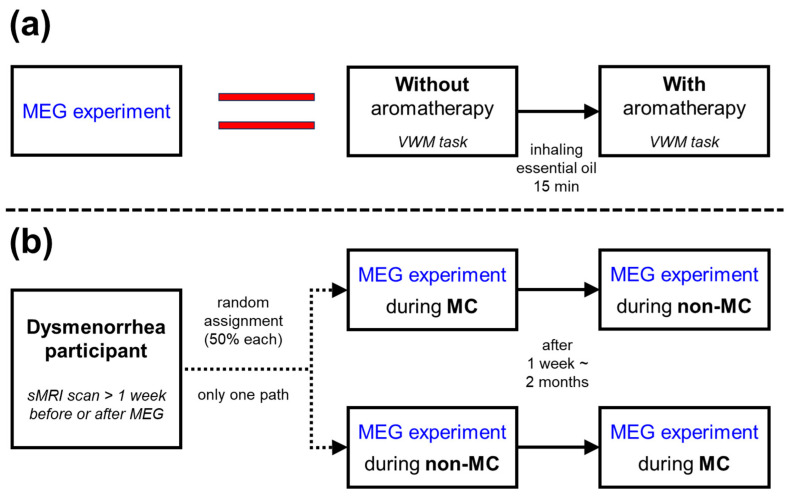
(**a**) The timeline flowchart of the MEG experiment comprises two VWM tasks: an initial VWM task without aromatherapy, followed by a 15 min period of Hinoki essential oil inhalation and a subsequent VWM task with aromatherapy. (**b**) The timeline flowchart for dysmenorrhea participants undergoing MEG experiments entails an sMRI scan one week before and after the MEG scan. Ultimately, each participant completes two MEG experiments: one during non-MC and another during MC. Participants are initially assigned to one of two experimental sequences: 50% are assigned to the “MC-first, non-MC-second” sequence, while the remaining 50% are assigned to the “non-MC-first, MC-second” sequence. The interval between the two MEG experiments ranges from one week to two months.

**Figure 2:**
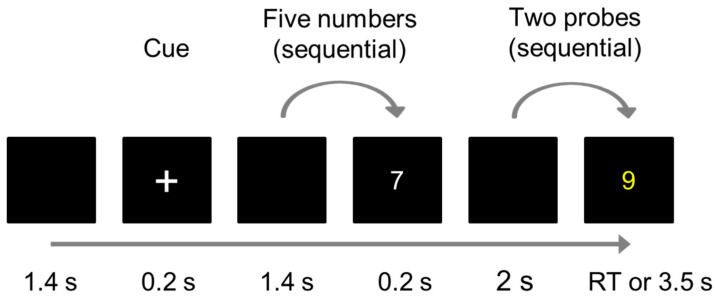
Design and protocols of the VWM test for each task (one cue, five numbers, two probes). Participants were instructed to memorize five unique, randomly generated white numbers. They were then required to answer whether each yellow probe number matched one of the previously presented white numbers (RT = reaction time).

Twenty-four female participants [mean age ± standard deviation, 22.17 ± 1.71 years; median (range), 22.5 (20–26) years] with lower-abdominal pain during menstruation, confirmed by visual analog scale (VAS) scores, were recorded in replicated measurements on separate days (please refer to the Supplementary File for participants’ characteristics). While not screened for academic background, participants were recruited based on self-reported absence of major medical history, including mental health conditions. The counterbalanced design study consisted of two separate sessions [MC and nonmenstruation (non-MC) periods] for each participant, and each measurement day included two repeated measurements (without and with aromatherapy) as shown in Figure 1. Each measurement consisted of a VWM task (including resting state), during which the participants experienced some mild physiological pain caused by their MC. First-time working memory participants had completed a pre-test practice session to familiarize themselves with the task before undergoing the experiment. The experimental protocol for each task is shown in Figure 2. A 1.4 s blank screen was followed by a 0.2 s fixation cross cue, indicating the beginning of the task. Subsequently, five non-repetitive randomly generated white numbers were presented for 0.2 s each, with a 1.4 s interval between each number. Participants were instructed to memorize those five numbers. Two yellow probe numbers were presented sequentially for 3.5 s or until the participant responded. A 2 s blank screen appeared before each probe number. Participants were required to answer whether each yellow probe number (question) matched one of the previously presented white numbers. All participants used their right index finger to press the match key and their left index finger to press the nonmatch key. The total measurement consisted of 80 working memory tasks and 160 match/nonmatch questions (2 questions per task). The ratio of match-to-nonmatch questions was 6:4. Neural activities were measured by MEG when the participants performed the working memory task.

#### Ethical Approval

This study was approved by the Institutional Review Board of Research Ethics committee of National Taiwan Normal University with the REC number 201607HM006 (Proposal title: The exploration of neural mechanism between menstrual pain and mind-brain interaction). The experiments were undertaken with the understanding and written informed consent of each participant.

Hinoki essential oil, a captivating essence extracted from the majestic Hinoki trees that tower over forests of Japan, Taiwan, and America, exudes a refreshing and calming aroma. Steam distillation reigns supreme as the most common method for extracting this precious oil. Hinoki aromatherapy was used as described follows. Hinoki essential oil (2 mL) was prepared in a smelling bottle and hung around the participant’s neck. The participant closed their eyes and relaxed while inhaling the essential oil for 15 min. Then, the resting-state and working memory tasks were performed, and the smelling bottle remained around the participant’s neck throughout the experiment.

### 2.2. Acquisition of MEG Data

MEG data were collected using a 306-channel Triux MEG system (Elekta Neuromag) based on SQUIDs in a magnetically shielded room at the Imaging Center for Integrated Body, Mind and Culture Research, National Taiwan University (Taipei, Taiwan). The helmet-shaped system consisted of 102 magnetometers and 204 gradiometers, arranged in a helmet-shaped Dewar and used to measure the magnetic field component perpendicular to the channel surface. The MEG was sampled at a rate of 1 kHz, and the recorded bandpass filter had cutoff frequencies of 0.03 and 330 Hz. To determine the position of the participant’s head within the helmet and calculate its location in the sensor coordinate system, four localization coils were attached to the participant’s head. Two coils were placed on the forehead, one on each side. The other two coils were placed behind the ears, one on each side. The magnetic field generated by these coils was recorded by the MEG system. The positions of the four localization coils and three anatomical landmarks (left/right preauricular points and nasion) were measured using a digitizer (Polhemus Fastrak, Colchester, VT, USA). More than 100 scalp points were also registered. This digitization process was performed to achieve coordinate transformation between the head and sensor spaces, crucial for matching with sMRI. Trigger signals corresponding to working memory stimuli were also sent to the MEG system’s parallel trigger box via the stimulus computer and recorded by the MEG system. These signals were used to align MEG data with the stimulus presentation times, allowing for the analysis of neural correlates of working memory. Electrooculography (EOG) and electrocardiography (ECG) were recorded using MEG-compatible electrodes to record eye movements, blinks, and heart activity, allowing for the removal of these artifacts in subsequent analyses.

### 2.3. Acquisition of sMRI

All participants underwent sMRI scans using a Siemens Magnetom Prisma (3T) scanner at the Imaging Center for Integrated Body, Mind and Culture Research, National Taiwan University (Taipei, Taiwan). The scan covered the entire head with a field of view of 192 × 192 mm and captured images with a high resolution of 1 × 1 × 1 mm. To minimize the potential confounding effects of MEG and sMRI scans, the influence of these scans on the results was carefully controlled and eliminated.

### 2.4. Data Analysis

Preprocessing of MEG data was performed using Elekta’s Maxfilter to remove environmental noise using a signal space separation method [24]. Processed data were analyzed using MNE to estimate the location of cortical neuronal activity [22]. sMRI was processed using the FreeSurfer software package (v6.0.0) to reconstruct the cortex and head model [25]. First, raw data were processed using MNE’s built-in independent component analysis function to remove eye movement and heartbeat artifacts from EOG and ECG channels. Raw data were bandpass-filtered between 1 and 45 Hz for sensor analysis (source analysis used 1–30 Hz and 8–12 Hz, respectively). Visual stimulus timing delays have been corrected. The appearance of the probe defined time 0 (stimulus onset) and the prestimulus interval was used to determine the baseline. Experimental data were segmented into epochs from 0.2 s before stimulus onset to 0.5 s after stimulus onset. Ninety-six trials (match questions) were averaged to improve the signal-to-noise ratio (SNR).

For source reconstruction, MRI (defining the source space and conductor model), the head (digitized four localization coils), and the MEG device (MEG sensors) need to be coregistered in the same coordinate system. The candidate dipoles were localized on the cortical mantle. A cortical source space was chosen, and the source space for each hemisphere was obtained by recursively subdividing the shape, resulting in evenly distributed dipoles on the brain surface for each participant. MNE uses a default conductivity of 0.3 S/m for the brain and scalp and 0.006 S/m for the skull. The BEM surface and source space were coregistered with MEG sensors, and dSPM was calculated using MNE default values: loose orientation of 0.2, depth weighting of 0.8, and SNR value of 3. Data of all participants were transformed into a common space to help compensate for individual differences and normalize the data. Data were morphed to the standard FreeSurfer average subject (fsaverage). After the morphing was completed, data were simply averaged.

### 2.5. Statistical Analysis

We compared the behavioral results and brain activation intensity of each participant after the working memory test. We investigated whether there were any differences in the accuracy (% correct), amplitude (activation intensity), and latency (reaction time) of working memory during the nonmenstrual and menstrual phases, with or without aromatherapy, for all participants.

Another statistical analysis used a cluster-based permutation test (implemented in MNE) on source time courses (dSPM) [23]. The null hypothesis was that there was no significant difference in data distribution without and with aromatherapy during the menstrual phase. Under each permutation, a paired *t*-test was performed for each participant at each source space vertex and time point to compare the absolute difference of the image data without and with aromatherapy during the menstrual phase. These differences were clustered, and the maximum cluster size from each permutation was selected to form the null distribution. The cluster size of the real data was compared to this null distribution, and the final goal was to find clusters that led us to reject the null hypothesis (*p* < 0.05).

## 3. Results

### 3.1. Hinoki Aromatherapy May Improve Working Memory Efficiency of Dysmenorrhea Participants during MC Based on Behavioral Performance

**Figure 3:**
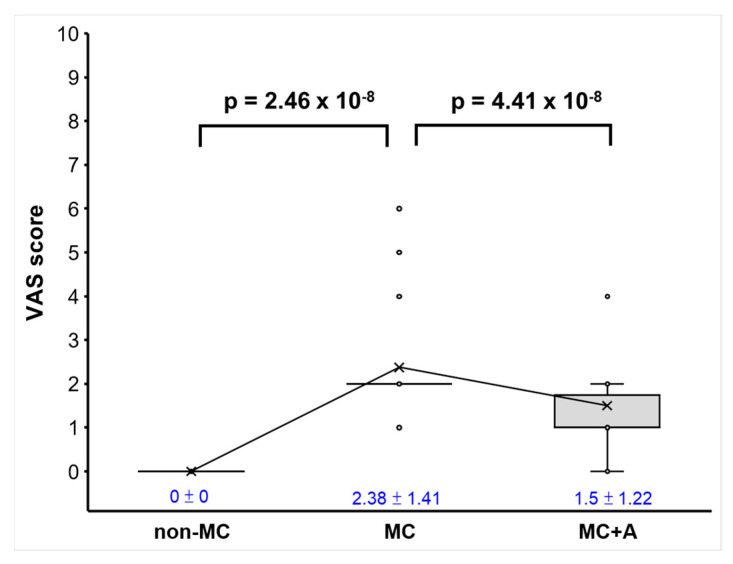
VAS score box plot (n = 24) reveals that most dysmenorrhea cases in the MC group were mild. The MC+A group experienced a significant reduction in pain. Mean ± standard deviation values are indicated in blue, and the trendline connects the mean values for each condition.

Figure 3 presents a box plot illustrating the VAS score for three groups: non-MC, MC, and MC+A (with aromatherapy). The graph indicates that the MC group experienced significantly higher VAS scores compared to the non-MC group (*p* = 2.46 × 10^−8^), suggesting a higher pain level in the MC group. While the MC group had a higher VAS score, it is worth noting that the majority of cases within this group were categorized as mild dysmenorrhea. Additionally, the VAS score for the MC+A group was significantly lower than that for the MC group (*p* = 4.41 × 10^−8^), implying that aromatherapy provided dysmenorrhea relief.

**Figure 4:**
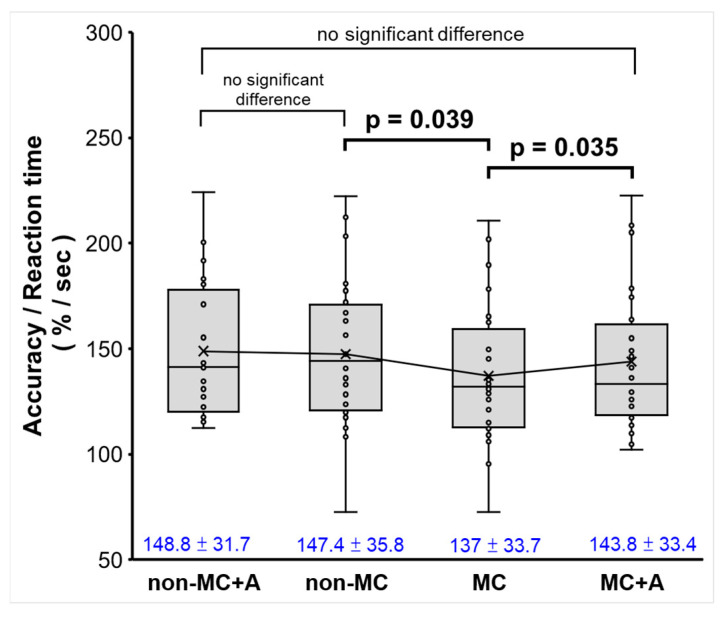
Accuracy/reaction time by MEG measurement in response to VWM from dysmenorrhea participants during non-MC and MC periods with or without aromatherapy (A). Data are the mean ± standard deviation, indicated in blue font (n = 24). *p* < 0.05 (paired *t*-test). The trendline shows the general tendency of the mean values across different conditions.

In Figure 4 (box plot), 24 participants completed the working memory test. The ratio of accuracy to reaction time (accuracy/reaction time) was analyzed. The results showed that the non-MC+A group (mean ± standard deviation, 148.8 ± 31.7) performed similarly to the non-MC group (147.4 ± 35.8), suggesting that aromatherapy did not influence non-MC performance. However, the MC group (137.0 ± 33.7) exhibited a lower ratio compared to both non-MC groups, indicating that MC affected the speed (reaction time) for the working memory task. Interestingly, the MC with aromatherapy group (143.8 ± 33.4) exhibited a higher accuracy/reaction time ratio than the MC without aromatherapy group, suggesting a potential benefit of aromatherapy in improving task efficiency during MC. However, this ratio remained lower than the non-MC group, indicating that even with aromatherapy, performance during MC did not fully recover to non-MC levels. Notably, there was no significant difference between the non-MC+A and MC +A groups. The MC+A group demonstrated significantly improved performance compared to the MC group, while the non-MC+A group showed a non-significant performance enhancement (slightly) compared to the non-MC group. This suggests that aromatherapy contributed to the improved performance of the MC+A group rather than the non-MC+A group, resulting in no significant difference between the non-MC+A and MC+A groups. A significant difference (*p* = 0.039) was observed between non-MC and MC groups without Hinoki aromatherapy, and another significant difference (*p* = 0.035) was observed between the MC groups without and with Hinoki aromatherapy. All groups had similar accuracy, but MC affected the speed (reaction time) for the working memory task. The task was not designed to detect differences in performance between groups, as it only had one difficulty level (five numbers). Data supported previous findings that cortical activation differences between groups are due to different activation levels at the same level of task difficulty rather than different task difficulty levels between groups [26].

### 3.2. VWM Task Activates Two Distinct Peak Intervals in MEG Signals

**Figure 5:**
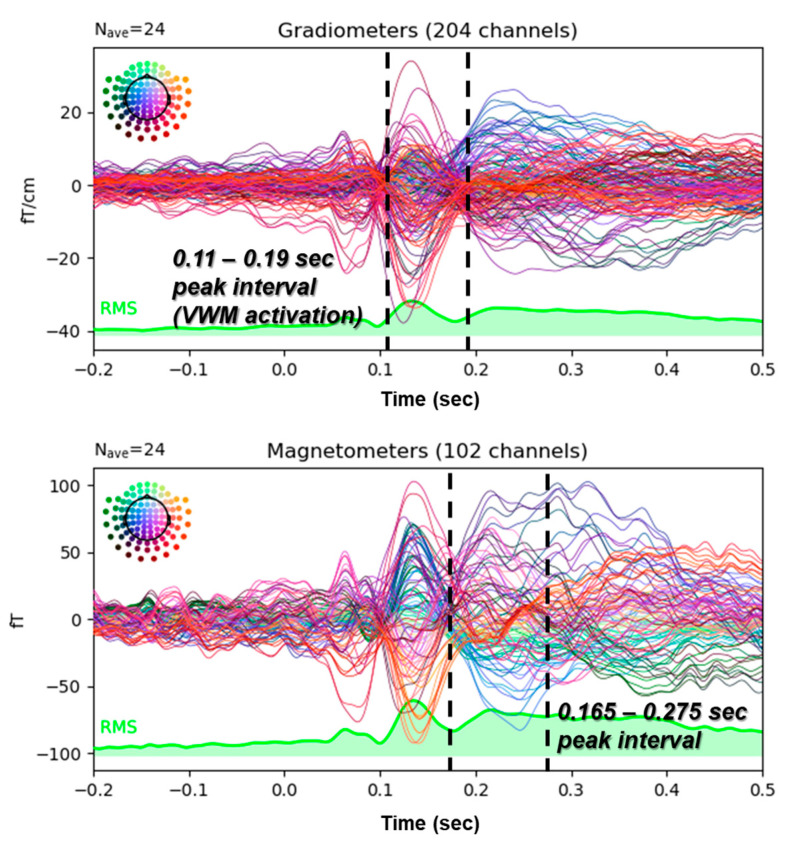
Grand average of the MEG signal in response to VWM from dysmenorrhea participants during MC with aromatherapy. All channels of the gradiometer and magnetometer, respectively, measure magnetic fields in units of femtotesla (fT), with positive and negative values indicating opposite magnetic field directions. The sensor signal peak intervals, denoted by the two dashed lines, are (0.11 to 0.19) and (0.165 to 0.275) s. These intervals play a crucial role in our subsequent permutation *t*-test on source data analysis.

All brain signals measured by the MEG system (averaged waveforms across multiple trials) allow us to preliminarily determine the time window with the greatest amplitude variation at the sensor level. This time window (sensor level) is then used for subsequent more detailed source localization (source level). Finally, the brain sources (source level) are subjected to a permutation *t*-test to generate statistical results. Therefore, this subsection focuses on the preliminary determination of the time window with the greatest brainwave amplitude at the sensor level. Figure 5 displays the grand average of the peak time for the strongest time-domain MEG sensor-level signal (1–45 Hz) during the VWM task across all participants. No significant difference in sensor-level intensity was observed between the MC and MC+A periods. The figure consists of two panels, the upper panel for gradiometers (measuring near-field brainwaves) and the other for magnetometers (measuring far-field brainwaves). Each line in the panel represents the brainwave signal recorded by specific sensors. The green RMS area below the panel represents the total energy across all sensors. The color-coded circles in the upper left corner of each panel indicate the locations of the corresponding sensors. The dark red and orange channels exhibit higher intensity (0.11 to 0.19 s), suggesting increased brain activity in the occipital-temporal region. The most likely source of the occipital-temporal peak is the fusiform gyrus [27]. The waveform of the sensor-level signal is similar to previous research [26]. Given the stimulus characteristics (numbers) and the total channel energy value, the most dominant peak interval occurred at 0.11 to 0.19 s (VWM activation), followed by another peak interval at 0.165 to 0.275 s after VWM activation. These two peak time intervals formed the primary focus of this study. The upcoming permutation *t*-test on source data will explore how aromatherapy’s ability to lower brain activity might activate brain regions involved in dysmenorrhea relief by targeting these time periods.

### 3.3. Permutation t-Test on Source Data Reveals Reduced Intensity in Pain Processing Regions (0.11–0.19 s) with Hinoki Aromatherapy during MC

**Figure 6:**
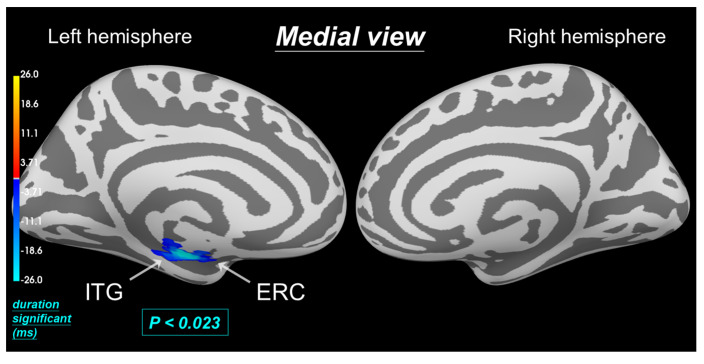
Spatiotemporal source space clusters showing significant differences in brain activity without and with aromatherapy during MC. Clusters represent brain regions (left ERC and ITG in the medial cortex) where the difference in activation intensity without and with aromatherapy during MC was statistically significant (*p* < 0.023). Blue indicates that the activation intensity was lower with aromatherapy than without aromatherapy over time (duration significant).

After filtering brain waves with a 1 to 30 Hz bandpass filter, dSPM intensity in specific brain regions was analyzed without and with aromatherapy during MC (please refer to the Supplementary File). dSPM analysis was performed using a permutation *t*-test on source data with spatiotemporal clustering, allowing statistically significant differences in activation intensity to be identified between the two conditions while controlling for multiple comparisons. Figure 6 shows the statistical analysis results, with blue indicating that the intensity with aromatherapy was lower than without aromatherapy, particularly within the 0.11 to 0.19 s window after the visual stimulus, and “duration significant (ms)” represents the duration of statistically significant differences when a *p*-value is specified (*p* < 0.023), where the maximum “duration” of significant differences is about 26 ms, with darker blue indicating shorter durations and lighter blue indicating longer durations. In addition, we initially set the *p*-value threshold at 0.05 and gradually adjusted it to 0.023 to obtain the smallest *p*-value with significant differences.

Previous research suggested that left ERC and ITG play a crucial role in pain processing in PDM, with studies showing a positive correlation between activation intensity in these areas and the severity of menstrual pain [5]. Therefore, the findings suggested that Hinoki aromatherapy administered after the onset of MC can alleviate dysmenorrhea. This was further supported by the observation that Hinoki aromatherapy significantly reduces dSPM intensity in both brain regions (*p* < 0.023) compared to no aromatherapy treatment during MC. This reduction in activation intensity, particularly within the 0.11 to 0.19 s window, may be a potential biomarker for pain relief associated with aromatherapy treatment.

**Figure 7:**
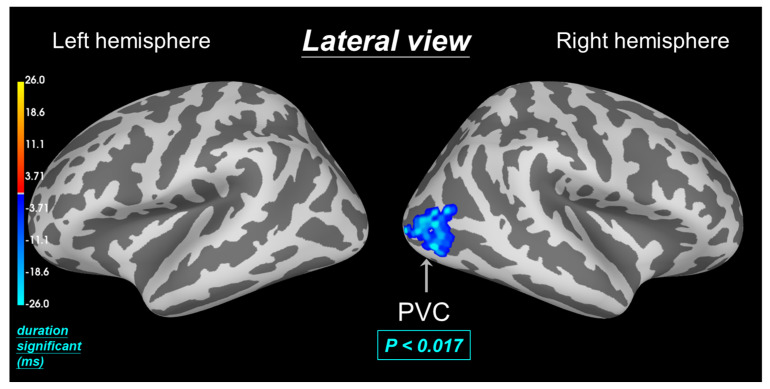
Aromatherapy-induced modulation of brain activity during MC is visualized through spatiotemporal source space clusters. These clusters highlight brain regions (PVC in the lateral cortex) that exhibited statistically significant changes in activation intensity (*p* < 0.017) following aromatherapy intervention compared to the without aromatherapy state. Blue regions signify a reduction in activation intensity with aromatherapy over time (duration significant).

Another result within the 0.11 to 0.19 s window (Figure 7) revealed a statistically significant difference, with blue indicating lower dSPM intensity with aromatherapy. Previous studies suggested that PVC is involved in pain processing in migraine and PDM [5,19]. The shared pathophysiology of menstrual migraine and dysmenorrhea, involving prostaglandin overproduction, further supported this connection [20]. This shared underlying mechanism suggested the potential for overlapping treatment strategies to address these coexisting pain syndromes. Findings indicated that dysmenorrhea-induced pain can influence VWM performance. Furthermore, a reduction in dSPM activation intensity in the visual cortex with Hinoki aromatherapy (*p* < 0.017) compared to no treatment suggested that aromatherapy may be a potential biomarker for pain relief associated with dysmenorrhea.

### 3.4. Permutation t-Test on Source Data Reveals Reduced Intensity in Pain Processing Regions (0.165–0.275 s) with Hinoki Aromatherapy during MC

**Figure 8:**
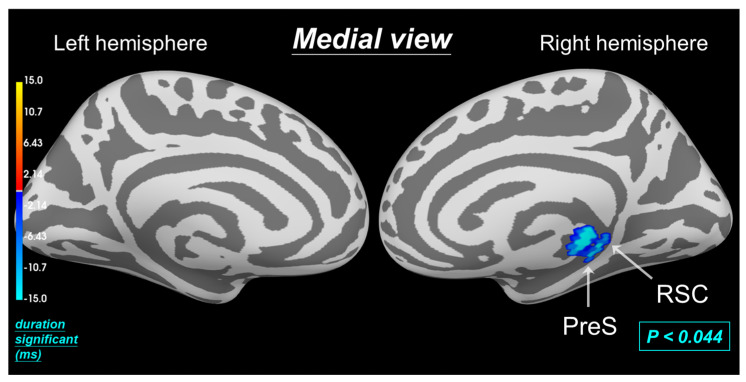
Visualizing the effects of aromatherapy on brain activity during MC: spatiotemporal source space clusters. These clusters represent brain regions (RSC and PreS in the medial cortex) where aromatherapy intervention elicited statistically significant (*p* < 0.044) changes in activation intensity compared to no aromatherapy intervention. Blue areas show a decrease in brain activity with aromatherapy over time (duration significant).

After filtering brain waves with an 8 to 12 Hz bandpass filter, a permutation *t*-test on source data with spatiotemporal clustering was employed to analyze dSPM intensity in RSC and PreS without and with aromatherapy during MC. In Figure 8, the analysis revealed a statistically significant difference, with blue indicating lower dSPM intensity with aromatherapy. Previous research suggested that RSC and PreS are also involved in PDM. RSC exhibited a positive correlation with pain intensity, whereas PreS connected the hippocampus and amygdala, suggesting its role in processing emotional memories related to pain [5,21]. These findings suggested that aromatherapy administered after the onset of MC can alleviate dysmenorrhea. This was further supported by the observation that Hinoki aromatherapy significantly reduces dSPM intensity in RSC and PreS (*p* < 0.044) compared to no aromatherapy treatment during MC.

## 4. Discussion

The main findings of this study are the differences in neuronal activation and physiological parameters during the execution of VWM tasks between non-MC and MC women. Neurons in the human inferior occipitotemporal cortex are selective for specific categories of visual stimuli, such as numbers, letters, and faces, within a short period after stimulation [27]. This study provides evidence that the latency of the working memory is ~0.11–0.19 s, and its activation intensity occurs in occipitotemporal lobes, which can represent cortical neuronal responses to VWM. Data further indicate that dysmenorrhea leads to a significant difference in the activation intensity of the visual area of working memory and causes significant changes in behavioral performance and brain areas related to dysmenorrhea.

MEG is a powerful tool for detecting human brain activity, especially neuronal activity. The principle is that neuronal signal conduction causes an electronic current that in turn generates a magnetic field. Therefore, the magnetic signal can be recorded by multichannel magnetic sensors to construct a magnetic field map outside the human brain. The acquisition frequency of MEG is typically 1 kHz, which provides a high temporal resolution of 1 ms, the same as EEG. The magnetic signal propagating from the brain to the magnetic sensor is not affected by the human head because the permeability of the human head is almost equal to μ0, the permeability in a vacuum. The magnetic field distribution outside the human head can resolve the current distribution inside the brain using various source localization methods, such as equivalent current dipole (ECD) or minimum-norm estimation. However, the electrical signal is affected by the human head. Therefore, the spatial resolution of MEG is several millimeters, much better than the ~1 cm spatial resolution of EEG. This allows us to detect significant differences in the activation intensity of the occipital-temporal region of the brain without and with aromatherapy, even in the medial cortex. Several studies have modeled the cortical sources of N170 (by EEG) and M170 (by MEG) working memory latency as ECDs. N170 sources have been localized to the fusiform gyri [28] or additional structures such as the lingual gyri [29]. Previous EEG studies have demonstrated different generators for N170 sources, such as the lateral occipitotemporal cortex or the superior temporal sulci [30]. The M170 source by MEG has been consistently localized in or close to the fusiform gyri [31], indicating a stable source or location by MEG measurement. Therefore, the dSPM source reduction in this study clearly indicates that dysmenorrhea aromatherapy may affect dysmenorrhea-related brain regions through the VWM-related brain regions.

Orthodontic metals and hair dyes can affect the MEG signal. Young female participants often show these two characteristics, which increases the difficulty of obtaining enough cases. Severe dysmenorrhea interferes with the daily life of some participants who cannot complete the entire experiment of excess painful interference during the MC period. To ensure the reliability of the normal distribution of the experiment, this study excluded participants with extreme factors, such as those who had no dysmenorrhea or severe dysmenorrhea, and only recruited a majority of participants with mild dysmenorrhea. The findings are therefore only applicable to young adults in their early twenties experiencing mild menstrual pain.

Clinically, people with chronic pain are frequently associated with poor memory and concentration. Working memory is impaired in chronic pain [32]. One previous systemic review and meta-analysis found that healthy controls had better performance in working memory, the number of correct responses, and reaction time; however, no physiological evidence in EEG supported the differences between chronic pain participants and controls during working memory tests [33]. Valentini et al. (2017) suggested that both performance on neuropsychological tests of memory and personality factors related to cognitive/emotional regulation may predict behavioral and neural patterns of nociceptive working memory in healthy individuals [32].

This study found no significant differences in accuracy rate and reaction time separately between nonmenstruating and menstruating women, possibly due to the simple design of the working memory test. However, when analyzing the ratio of accuracy to reaction time without and with aromatherapy for dysmenorrhea, there was a significant difference. Behavioral data align with findings in the dysmenorrhea brain area study. This suggests that the participants’ response patterns, such as answering slowly but accurately or answering quickly but less accurately. Therefore, taking the ratio of accuracy to reaction time can eliminate this factor and should be able to address the issue of the task being too simple, enhancing task sensitivity. Aromatherapy for dysmenorrhea reduces the signal in these brain regions, potentially impacting the decrease in the visual dysmenorrhea signal. This suggests that pain reduction through aromatherapy may enhance VWM performance. This implies that pain may affect a neural pathway, potentially DPMS, which couples with these dysmenorrhea-related brain areas. The offset of the working memory peak latency may affect short-term memory performance.

Dysmenorrhea, although not a disease, is a recurring issue that can cause discomfort and interfere with concentration during MC. In the medical field, aromatherapy has been explored as a potential treatment for dysmenorrhea, but its efficacy and optimal use still require further research. Song et al. demonstrated that aromatherapy effectively reduces dysmenorrhea [16]. However, their analysis, which involved a systematic review and meta-analysis, revealed that the methods used for aroma intervention are highly diverse and lack a strong scientific basis. The study found a high likelihood of randomization bias. Another systematic review found moderate evidence that aromatherapy (inhalation, massage, and oral use) can effectively alleviate pain associated with PDM [17]. Inhalation of lavender has been reported to relieve the severity of PDM symptoms [15].

In this study, the combination of MEG and MRI clearly delineated the potential effect of Hinoki aromatherapy on spatially and temporally neurophysiologic alterations in PDM. Notably, the five brain regions identified as showing differences with aromatherapy during MC are all implicated in pain processing mechanisms, according to the existing literature. Additionally, our working memory experiment analysis using “match trials” suggests that participants were actively engaged in memory-related brain regions. This potential mechanism may be mediated by a “memory-pain” association. We propose that working memory acts as a mediator, concentrating brainwave energy in specific time ranges, while aromatherapy’s pain-reducing mechanisms (lowering brainwave intensity) induce these dysmenorrhea-relief brain regions.

However, prior to definitively validating aromatherapy as a successful intervention, it is essential to identify neurophysiological biomarkers associated with its effectiveness. This study focused on the immediate effects of Hinoki aromatherapy on working memory and neurophysiological responses, without considering the time factor. Future research could extend the investigation to examine the long-term effects of Hinoki aromatherapy on working memory and neurophysiological responses, as well as explore its efficacy in individuals with different types or severities of dysmenorrhea.

## 5. Conclusions

This study suggested that Hinoki aromatherapy may offer relief from dysmenorrhea and related symptoms by reducing activity in brain regions associated with pain processing. This aligns with emerging research highlighting specific brain regions as promising biomarkers of dysmenorrhea relief (as mentioned in the introduction section). Several studies have identified the involvement of the left ERC and ITG in physiological pain processing and its emotional aspects in dysmenorrhea [18]. Similarly, the PVC has been linked to dysmenorrhea through its role in DPMS in migraine [5,19]. This link is further supported by the shared mechanism of migraine and dysmenorrhea, involving prostaglandin overproduction, and the frequent co-occurrence of these conditions [20]. Additionally, the RSC and PreS show connections to pain intensity and the processing of emotional memories associated with PDM [5,21]. The observed decrease in activity within these areas with aromatherapy use in our study aligns with their potential as biomarkers of dysmenorrhea relief. These findings further strengthen the growing body of evidence supporting the use of aromatherapy as a complementary therapy for dysmenorrhea.

## Data Availability

Access to the datasets utilized in this study can be granted upon request to the corresponding author. However, due to privacy and ethical considerations, the data is not publicly accessible.

## Supplementary Materials

The following supporting information can be downloaded at: https://www.mdpi.com/article/10.3390/biomedicines12102189/s1, Figure S1: The grand average of dSPM values for all participants; Table S1: Original data table of age, height, weight, and VAS scores for all participants.

## Abbreviations

MEG	magnetoencephalography
MRI	magnetic resonance imaging
sMRI	structural MRI
VWM	visual working memory
MC	menstruation
MC+A	menstruation with aromatherapy
non-MC	nonmenstruation
non-MC+A	nonmenstruation with aromatherapy
PDM	primary dysmenorrhea
PAG	periaqueductal gray matter
PFC	prefrontal cortex
BDNF	brain-derived neurotrophic factor
DPMS	descending pain modulatory systems
ERPs	event-related potentials
EEG	electroencephalography
ERFs	event-related fields
ERC	entorhinal cortex
ITG	inferior temporal gyrus
PVC	primary visual cortex
RSC	retrosplenial cortex
PreS	presubiculum
dSPM	dynamic statistical parametric mapping
VAS	visual analog scale
EOG	electrooculography
ECG	electrocardiography
SNR	signal-to-noise ratio
ECD	equivalent current dipole
