# Supplementary material for: Potential Biomarkers of Dysmenorrhea Relief: A MEG Study of Hinoki Aromatherapy and Working Memory
Source: Biomedicines. 2024 Sep 26;12(10):2189. doi: 10.3390/biomedicines12102189 (PMC11504012; doi:10.3390/biomedicines12102189)
Supplement: Supplementary file 1 [file biomedicines-12-02189-s001.zip › biomedicines-3012020-supplementary.pdf]

Supplementary file:

Figure S1. The grand average of dSPM values for all participants.

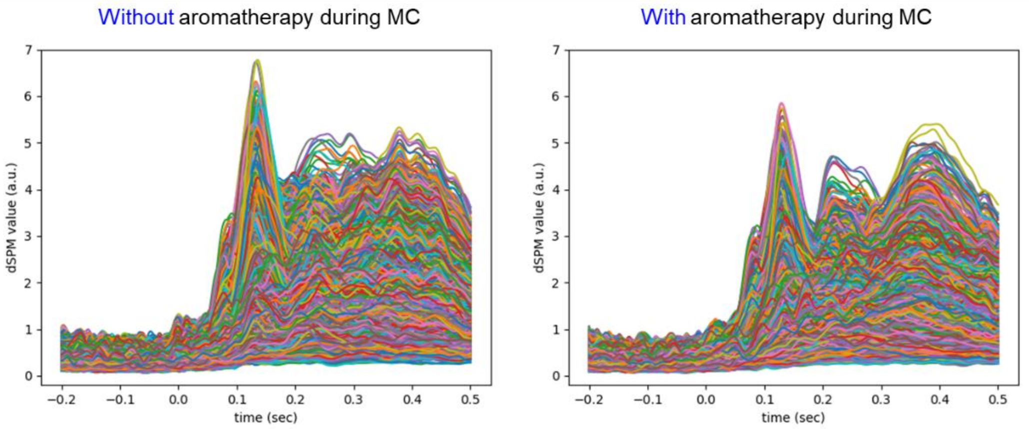

Table S1. Original data table of age, height, weight, and VAS scores for all participants.

Participant ID	Age (years)	Height (cm)	Weight (kg)	VAS score without aromatherapy during MC	VAS score with aromatherapy during MC
**1**	23	152	40	2	2
**2**	22	168	43	4	4
**3**	21	156	46.5	6	4
**4**	21	156	46	2	1
**5**	20	155.5	51	2	1
**6**	20	153	51	2	1
**7**	20	160	52	1	1
**8**	23	152	40	1	0
**9**	23	155	45	2	1
**10**	23	165	58	2	1
**11**	22	161	55	1	1
**12**	20	157	46	2	1
**13**	20	160	55	2	1
**14**	23	158	46	1	0
**15**	23	160	49	2	1
**16**	24	160	46	2	1
**17**	24	160	60	2	1
**18**	26	162	50	2	1
**19**	23	157	46	5	4
**20**	25	158	57.5	2	1
**21**	20	175	60	6	4
**22**	23	159	57	2	2
**23**	22	160	54	2	1
**24**	21	159	44	2	1
**Mean**	**22.17**	**159.10**	**49.92**	**2.38**	**1.50**
**SD**	**1.71**	**5.03**	**6.06**	**1.41**	**1.22**
**Median**	**22.5**	**159**	**49.5**	**2**	**1**
**Range**	**20 - 26**	**152 - 175**	**40 - 60**	**1 - 6**	**0 - 4**
